# Fertility differential of women in Bangladesh demographic and health survey 2014

**DOI:** 10.1186/s40738-017-0043-z

**Published:** 2017-10-13

**Authors:** Shongkour Roy, Sharif Mohammed Ismail Hossain

**Affiliations:** Population Council, Dhaka, Bangladesh

**Keywords:** Fertility, BDHS, Women

## Abstract

**Background:**

The aim of this study was to examine the fertility differential of women age 15 to 49 using data from Bangladesh Demographic and Health Survey 2014- a survey of women who were born from 1963 to 1999.

**Methods:**

The secondary data analysis was carried out using the BDHS 2014 in order to discuss differences in childbearing practices in Bangladesh. Descriptive statistics were used to analyze the data including education level, geographic location, and religion. A trend test used to assess the inferences.

**Results:**

On average, women had 2.3 children in the BDHS 2014; more than 90% of them gave birth to at least one child by age 49 and the average age of first birth was 18 years. Fertility of women strongly differed by education (*p* < 0.001). The percentage of women with secondary education who had no child was 50.3% and never attended school 8.4%;those with secondary education were six times as likely as those who never attended school to have no child and this pattern was stronger among urban compared with rural women.

**Conclusions:**

Fertility differential becomes robust as education increases. Women’s fertility is also related to religion and residence, but these factors were not strongly related as those educational attainments.

## Background

Fertility is the major component of population dynamics that decide the size, structure, and composition of populations in any country of the world [1]. Fertility behaviors have changed in Bangladesh since 1975. Research has shown that women childbearing decsions are impacted by the increase in universal provision of family planning services, and rates of women’s educational attainment and urbanization. Differentials in fertility behavior and fertility levels in different population strata have been the most pervasive findings in the demography [2, 3]. Bangladesh has seen large improvements in reproductive health, such as reducing maternal and infant mortality, increases in contraceptive prevalence and health service use among married women [1].

The Total Fertility Rate (TFR) in Bangladesh was dropped markedly from 6.3 births each women in 1975 to 2.3 births each women in 2014. But, TFR was still high compared to other developing countries. Ahamed [4] examined differentials of fertility with selected demographic and socioeconomic characteristics for ever-married women and their husbands. He showed that a woman’s age has the highest effect on change in fertility. Ramesh [5] found that demographic, socioeconomic, and cultural causes affect fertility differentials in Nepal. Age at first marriage of women was a strong predictor of ever having children. Desalew Zelalem conducted a study about the level and patterns of fertility among women using the Kersa Demographic Surveillance [6]. This author used follow-up data 2008–2012 and found that the fertility rate was higher in rural illiterate women than urban literate women. Kasey [7] and Aughinbaugh [8] used the National Longitudinal Survey of Youth 1979 (NLSY79) to examine the wage-earning implications of delaying the first birth. This article found that an annual 3 % wage premium existed for each year of delayed motherhood. Delayed childbirth also correlated with high test scores, education, and professional status of the mother [9–11].

In the Bangladesh Demographic and Health Survey (BDHS) 2014, early analyses were summary statistics with different characteristics such as marriage and sexuality, fertility preferences, fertility control, child health, nutrition, and female empowerment. However, they did not analyze this data for differences in childbearing practices and relationships with demographic factors. Our study focuses on differences in fertility patterns by educational attainment (no education, primary, secondary and higher), religion, and residence. Primary education is defined at completing grade 1 to 5, secondary education 6 to 10 grade, higher education 11 to 17 grade, and no education was those who never attended school. The analysis of this study is descriptive and does not try to discuss why fertility patterns differ among women.

## Methods

To examine fertility differential change, this paper has used data from the Bangladesh Demographic and Health Survey (BDHS) 2014. Details of the study design and data collection methods have been previously published [1]. Briefly, BDHS 2014 was a cross-sectional study designed to obtain information on key health indicators in Bangladesh i.e. fertility, family planning, maternal and child health, and women. The data used in this study is publicly available.

We conducted a secondary data analysis which examined fertility trend and differentials. Univariate and bivariate analyses have been performed to find out the sample characteristics and evaluate the differences in fertility patterns by following factors: educational attainment, religion, and residence. We also used a trend test to detect significant fertility differences.

## Results

A total of 17,863 women participated in the BDHS 2014 between ages 15–49. 29.5% women were young whose age group 15 to 24. A rural area is defined by Upazilla and is divided into Union parishads and, within Union parishads, into Mouzas. Most women lived in rural areas (71.7%). Participants’ higher education level had 8.5%, 37.4% had secondary, and 24.9% had no education. At most one tenth participants were non-Muslim and 10.2% had no child. Among the participants, 29.3% had two children and 7.8% had five or more children. The wealth quintiles in BDHS 2014 are a composite measure of a household’s cumulative living standard. The wealth quintiles are calculated using data on a household’s ownership of selected assets, such as televisions and bicycles; materials used for housing construction, and types of water access and sanitation facilities. More than one-fifth of the population (21%) were in the richest cohort of women (see, Table 1).

**Table 1:** Background characteristics [*N* = 17,863]

Characteristics	N(Percent)
Age (Year)	
15–19	2023 (11.4)
20–24	3161 (18.1)
25–29	3343 (18.9)
30–34	3012 (17.1)
35–39	2340 (12.9)
40–44	2170 (11.7)
45–49	1814 (9.9)
Education	
No education	4206 (24.9)
Primary	5226 (29.2)
Secondary	6722 (37.4)
Higher	1709 (8.5)
Religion	
Muslim	16,135 (90.1)
Non- Muslim	1727 (9.9)
Residence	
Urban	6167 (28.3)
Rural	11,696 (71.7)
Parity	
No Child	1891 (10.2)
One child	4247 (23.8)
Two children	5193 (29.3)
Three children	3353 (19.1)
Four children	1769 (9.7)
Five or more children	1410 (7.9)
Wealth Index	
Poorest, Q1	3251 (18.8)
Poorer, Q2	3360 (19.1)
Middle, Q3	3621 (19.9)
Richer, Q4	3769 (21.0)
Richest, Q5	3862 (21.2)

Note 1: Rural area is define as an area where an Upazila is divided into Union parishads and, within Union parishads, into Mouzas; Urban area is define as an area in Paurosova and words

Note 2: Wealth quintiles is define as a composite measure of a household’s cumulative living standard. The wealth quintiles in BDHS 2014 were calculated using data on a household’s ownership of selected assets, such as televisions and bicycles; materials used for housing construction; and types of water access and sanitation facilities

**Fig. 1:**
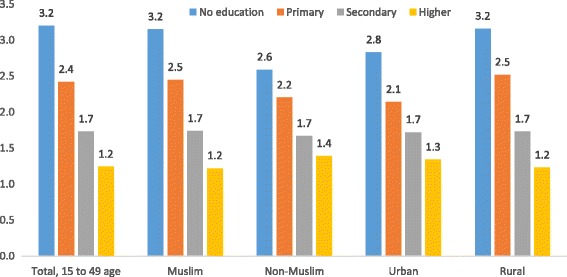
Mean number of children with education levels and demographic factors

Table 2 shows the fertility outcomes of women age from 15 to 49, by educational attainment, religion, and residence. Among women with higher education, 19.9% have no child, 13.8% have one child, 8.5% have two children, 3.3% have three children, 0.9% have four children and 0.4% have five or more children. Of women with secondary education, 50.3% have no child, 49.9% have one child, 41.9% have two children, 28.2% have three children, 19.3% have four children, and 10.8% have five or more children. On the other hand, among women with no education, 8.4% have no child, 11.9% have one child, 20.1% have two children, 34.0% have three children, and 45.4% have four children and 56.4% have five or more children. Thus, examining the fertility pattern with education disclosed a key significant differences across the groups (*p* < 0.001). Non-Muslim more educated women were less likely to have five or more children compared with uneducated women (0.01% versus 48.1%, *P* < 0.001) and Muslim women with no children at the highest education had also significant differences (20.2% versus 8.3%) in the lowest education (*P* < 0.001). Minority groups were over-represented among those with secondary educational attainment and under-represented among that higher education. Urban women were more likely to have no children compared with rural women (11.7% versus 9.6%).Rural women were more likely to have five or more children than urban (8.9% versus 4.8%). The mean number of children was 2.23 in BDHS 2014, those with women having no education having the highest mean number of children (3.2) and the lowest (1.2) incidence of higher education (see, Fig. 1).

**Table 2:** Fertility outcomes of women from age 15 to 49, by educational attainment, religion and residence

Characteristics	Percent distribution of people by number of children						Trend Test *p*-value [Lowest to highest education level]	Mean number of living children
	No child	One child	Two children	Three children	Four children	Five or more children		
N	1891	4247	5193	3353	1769	1410		17,863
Total, 15 to 49 age	10.2	23.8	29.3	19.0	9.9	7.8		2.23
No education	8.4	11.9	20.0	34.0	45.4	56.4	ref	3.2
Primary	21.4	24.4	29.6	34.5	34.5	32.4	*P* < 0.001	2.4
Secondary	50.3	49.9	41.9	28.2	19.2	10.8	*P* < 0.001	1.7
Higher	19.9	13.8	8.5	3.3	0.9	0.4	*P* < 0.001	1.2
N	1726	3807	4584	3035	1627	1356		16,135
Muslim	10.3	23.6	28.5	19.2	10.1	8.3		2.25
No education	8.3	11.9	19.2	33.3	45.0	56.7	ref	3.2
Primary	20.7	24.9	30.5	34.9	34.5	32.0	*P* < 0.001	2.5
Secondary	50.8	50.0	42.4	28.6	19.6	10.9	*P* < 0.001	1.7
Higher	20.2	13.2	7.9	3.2	0.9	0.4	*P* < 0.001	1.2
N	165	440	609	317	142	55		1728
Non-Muslim	8.9	25.1	36.1	19.1	8.2	2.6		2.0
No education	9.1	11.5	26.4	40.6	49.1	48.1	ref	2.6
Primary	28.3	20.5	23.1	30.6	34.6	43.7	0.010	2.2
Secondary	45.6	49.1	38.3	25.3	15.6	8.2	*P* < 0.001	1.7
Higher	17.0	18.9	12.2	3.5	0.7	0.0	*P* < 0.001	1.4
N	715	1666	1877	1076	502	331		6167
Urban	11.7	27.8	30.5	17.4	7.8	4.8		1.9
No education	6.9	10.8	14.9	27.9	43.1	55.9	ref	2.8
Primary	23.3	21.9	23.7	31.2	31.7	27.9	*P* < 0.001	2.1
Secondary	42.9	45.4	44.2	32.6	23.7	15.3	*P* < 0.001	1.7
Higher	26.9	21.9	17.2	8.3	1.5	0.9	*P* < 0.001	1.3
N	1176	2581	3316	2277	1267	1079		11,696
Rural	9.6	22.2	28.8	19.8	10.7	8.9		2.7
No education	9.1	12.5	22.2	36.1	46.0	56.5	ref	3.2
Primary	20.5	25.6	32.1	35.7	35.2	33.4	*P* < 0.001	2.5
Secondary	53.9	52.2	40.9	26.7	17.9	9.8	*P* < 0.001	1.7
Higher	16.5	9.7	4.8	1.5	0.7	0.3	*P* < 0.001	1.2

Table 3 also presents descriptive statistics on women’s age and birth spacing children, educational attainment, religion, and residence. More educated women had children at the later ages. The average age at first birth was 19.0 years for women with one child, 18.0 years for women with two children, and 17.2 years for women with three children. The births of women in the latter group were spaced more closely than those of women with two children. On average, the time between first and second births were 5.7 years for women with two children, compared with 4.7 years for those with three children. Evaluation of birth spacing in women with no education by two and three children-women, found the mean time between first and second births to be 6.2 years for women with two children, compared with 4.9 years for those with three children.

**Table 3:** Women age at birth and birth spacing by number of children, educational attainment, religion and residence

Characteristics	All	Education				Religion		Residence	
		No education	Primary	Secondary	Higher	Muslim	Non- Muslim	Urban	Rural
Among those with one child									
Average age at first birth	19.0	18.9	17.9	18.5	22.3	18.9	20.1	19.7	18.5
Among those with two children									
Average age at first birth	18.0	17.5	17.1	17.9	21.9	17.9	18.8	18.6	17.7
Average age at 2nd birth	23.7	23.7	22.8	23.4	27.5	23.6	24.4	24.5	23.2
Year between 1st and 2nd child	**5.7**	**6.2**	**5.7**	**5.5**	**5.6**	**5.7**	**5.6**	**5.9**	**5.5**
Among those with three children									
Average age at first birth	17.2	17.0	16.9	17.5	20.9	17.2	17.7	17.5	17.1
Average age at 2nd birth	21.9	21.9	21.4	22.1	25.4	21.9	22.3	22.1	21.8
Average age at 3rd birth	26.8	26.9	26.2	26.9	30.2	26.8	26.8	27.2	26.6
Year between 1st and 2nd child	**4.7**	**4.9**	**4.5**	**4.6**	**4.5**	**4.7**	**4.7**	**4.6**	**4.7**
Year between 2^n^ and 3rd child	**4.9**	**5.0**	**4.8**	**4.8**	**4.8**	**4.9**	**4.5**	**5.1**	**4.7**
Year between 1st and 3rd child	**9.6**	**9.9**	**9.3**	**9.4**	**9.3**	**9.6**	**9.2**	**9.7**	**9.5**

Inferences: all boldface are significant at 0.1% level of significance

There were also birth spacing differences related to higher education,, residence, and religion. Education strongly correlates with age at first birth. Women with a higher education tend to have first birth at older ages. These ages are 22.3 for women with one child, 21.9 for women with two children, and 20.9 for women with three children. At first birth, for a given number of children, women with a higher education are 3.9 to 4.9 years older than those with primary education and 3.4 to 4.0 years older than those with secondary education.

However, the age at first birth for urban women falls more steeply as total fertility increases. On average, urban women who have only one child are 19.7 years old at the birth of their child. Urban women who have two children are 18.6 and three children are 17.5 at their first birth. Moreover, on average, rural women who have only one child are age 18.5 at the first birth of their child and women who have two children are 17.7 and three children are 17.1 at their first birth.

## Discussion

The major finding of our study are that education and residential area impact the fertility of women age 15–49. More than 90% of women in the BDHS 2014 had at least one child by age 49 and 2.3 children was the average in this cross-sectional data set. Women from 15 to 49 who had higher education had the lowest fertility compared with women with only secondary, primary and no education, which means that fertility was delayed as education increased in Bangladesh.

These findings are similar to a previous study of demographic, socioeconomic, and cultural factors affecting fertility differentials in Nepal [5] which found strong relationships between fertility and demographic factors. Bangladesh, however, is quite similar to Nepal with regard to cultural activities and socioeconomic status. Globalization and regionalization supported by the revolution in technology have changed the socioeconomic landscape.

We was also found that the average age at first birth in urban areas was older, and that urban dwelling women were more likely to have lower fertility than rural women.

The present study has some limitations. The study included subjects from BDHS 2014. We did not include subjects from Sample vital registration system (SVRS). This population may not be representative of all classes of women in Bangladesh. The analysis in this study is only descriptive and did not try to explain why fertility patterns differ among women.

## Conclusions

Fertility patterns mostly differ by educational attainment rather than other factors, i.e. religion and residence. The mean number of living children in women with higher education is lower than women with less education. Fertility rates are higher in rural women than urban women with no education. Overall, women in rural areas and those with less advanced levels of education had more children, and women with higher education were older at the age of birth of their first child. We conclude that the education level contributes most to fertility differential in Bangladesh.

## Abbreviations

BDHS: Bangladesh demographic and health survey
NIPORT: National institute of population research and training
NLSY79: National longitudinal survey of youth 1979
Q1: First quantile
Q2: Second quantile
Q3: Third quantile
Q4: Fourth quantile
Q5: Fifth quantile
SVRS: Sample vital registration system
TFR: Total fertility rate
